# Croton Chloral*Read at the evening meeting of the Liverpool Chemists’ Association, February 12, 1874, and published in the *Chemist and Druggist*.

**Published:** 1874-05

**Authors:** Alfred H. Mason


### MISCELLANEOUS.

### Croton Chloral.*

BY ALFRED H. MASON, F. C S.

A new remedy, with chloral as its basis, and introduced by the discoverer of the therapeutical application of hydrate of chloral, naturally commands attention. At one of our general meetings in 1872 session, I exhibited a specimen of this, then new, compound, named by Professor Liebreich croton chloral hydrate.

Within the last few months this medicine has commanded much more of the attention of medical men, so that the requirements of it somewhat exceed the first demand for its predecessor when sold at about the same price.

Crotonic chloral was discovered somewhat accidentally by Dr. Kraemer and Dr. A. Pinner. These gentlemen were undertaking experiments on the action of chlorine on aldehyde, chiefly in the hope of thus obtaining chloral, and of being able to utilize the valueless residue from the first runnings obtained in the distillation of crude spirit, which consists mainly of alcohol, aldehyde, and paraldehyde.

Chlorine was passed into aldehyde, at first carefully cooled in a freezing mixture, and only heated to 100° at the close of the reaction. The first few bubbles caused the separation of a small quantity of solid metaldehyde, whether originally present in the aldehyde, or formed by the reaction, is undecided. After a short time evolution of hydro-chloric acid set in and every trace of chlorine was absorbed. With 100 grains of aldehyde, at the end of twenty-four hours, no further absorption took place even at 100°. The resulting brown mass consists of two layers: a lower, darker, almost solid; and an upper, lighter colored, liquid layer. The latter is a saturated solution of hydro-chloric acid and the bodies of the lower layer in water. As it was found impossible to separate these two well, the whole was submitted to distillation. A considerable quantity passed over between 90° and 100°; the thermometer then rose rapidly to 160°, and the main product distilled over between this and 180°; the temperature again rose to about 240°, but only decomposition products were obtained, and a considerable carbonaceous residue remained in the flask. By means of fractional distillation the portion boiling at 160° to 180° was quickly purified, and a body boiling at 163° to 165° was isolated, which proved to be crotonic chloral.

The specimen I have here was produced by passing perfectly dry chlorine gas over pure aldehyde ($C_2H_4O$)—the action is very

* Read at the evening meeting of the Liverpool Chemists' Association, February 12, 1874, and published in the *Chemist and Druggist.*

violent, and many precautions have to be taken to prevent explosion and to condense the volatile products of the reaction, and still to allow the enormous quantities of hydrochloric acid gas to escape. After a time the liquid thickens; at this stage the current of chlorine can be passed through the liquid. After another interval it becomes necessary to warm, and at last to boil the liquid through which the chlorine is passing. At length hydrochloric acid ceases to be evolved, and crude croton chloral is obtained—the process taking about forty-eight hours to complete. This crude body is *mainly* ordinary chloral, but mixed with a variety of other products. By fractional distillation and treatment with sulphuric acid—true croton chloral ($C_4H_3Cl_3O$)—trichlorcrotonic aldehyde is obtained. This is a dense oily liquid of peculiar odor, somewhat recalling ordinary chloral: treated with a considerable excess of warm water it hydrates and dissolves, and upon cooling, croton chloral hydrate ($C_4H_3Cl_3O$, $H_2O$) is deposited, but still in a crude form, most rank and offensive in flavor. It has to be purified by rather a tedious process, and is obtained, when pure, in beautiful white silvery crystals, with a sweetish melon flavor, which melt at 78 C.

From this it will be quite evident (and it is probably wise to note it) that this body does not bear any relation to croton oil, or crotonic acid, obtained therefrom, although its chemical constitution proves it to be the chlorated aldehyde of crotonic acid.

Croton chloral is the substance represented by the same term in the allyl ($C_3H_5$) group that choral has in the ethyl ($C_2H_5$) group. Its outward appearance differs from hydrate of chloral by the salt being much lighter, and in flocculent silvery crystals—by its being almost insoluble in cold water and very soluble in alcohol: it is soluble in hot distilled water, and rendered more easily so by the addition of 25 per cent. of pure glycerine; it is insoluble in chloroform.

It will be remembered that hydrate of chloral owes its value as a medicinal agent to the supposed elimination of chloroform when it comes in contact with the alkalies of the blood, it having been shown that by reaction with alkalies chloroform is produced. Crotonic chloral, when subject to the influence of an alkali, first forms allyl-chloroform, a trichlorated body which is rapidly decomposed into a bichlorated substance called bichlorallylene. In a communication to the *British Medical Journal*, December 20, 1873, Dr. Leibreich says:—"Both chloroform and trichlorated substances act in the first stage upon the brain; in the second, on the spinal cord; in the third, on the heart."

Although Dr. Leibreich's theory has met with and still finds general favor, there are many medical men who think it has not any valid support, believing that chloral exercises a specific action of its own upon the organization, which is not to be reasoned out from an exclusively chemical basis.

The medicinal advantages of hydrate of croton chloral over ordinary hydrate of chloral are:—1st. In cases where hydrate of chloral is inapplicable on account of heart disease (it does not interfere with the action of the heart. 2d. In cases of neuralgia in the district of the nervous trigeminus (it is a remarkable phenomenon that when given in small doses it produces anæsthesia of the fifth nerve, singling out one nerve, and that one alone, while the sensibility of the body generally and pulse and respiration remain unaffected). 3d. In cases where very large does are necessary to produce sleep, here Leibreich recommends the addition of croton chloral to hydrate of chloral.

Dr. Burney Yeo, of King's College Hospital, London, etc., is making a systematic investigation on the value of this medicine, and he lays his first communication in a paper published in the *Lancet*, January 31, 1874; he administered it in six different classes of cases, and gives details of each. The results he has arrived at are, that in croton chloral we possess a remedy of remarkable efficacy in some cases of neuralgia of the branches of the nervous trigeminus, and that it also has the power of affording relief in other obstinate forms of neuralgia; that it is of use in certain cases of diffused muscular pain; that there is scarcely any remedy that is likely to prove more valuable for the relief of the distressing night cough of chronic phthisis. Its efficacy in procuring sleep seems very variable in moderate doses; its effect in purely rheumatic cases is scarcely appreciable, while for hysteria it is of little or no use.

Dose.—Dr. Yeo says:—"I am satisfied that in dealing with this substance we must give an unusually wide range to the dose, for its effects vary greatly. The doses I have given varied from 1 to 10 grains. In delicate females I have found very decided effects from doses of 2 and 3 grains; in strong males a dose of ten grains is often required to produce any appreciable effect. As may be expected, persons who have been accustomed to the use of anodyne medicines require larger doses than others."

The dose must always be proportionate to the severity and long continuance of the pain. I would advise that it should be always given in moderate and quickly repeated doses, until the amount of "tolerance of the medicine in each particular case has been discovered. In severe neuralgias, from 2 to 5 grains may be given every hour, or the smaller dose every half hour, until 15 grains have been taken. At present I do not think it safe to go beyond this dose."

I have made several experiments with different solvents to present this medicine in a convenient form for dispensing, and before seeing Dr. Yeo's paper I found that the addition of glycerine was of great assistance in making the solution. I can fully endorse his decision. The following formulary yields the strongest solution that is permanent:—

| | | |
|---|---|---|
| Croton Chloral Hydrate | 64 | grains. |
| Pure Glycerine | ½ | ounce. |
| Hot Distilled Water | 1½ | " |

A syrup can be made containing 2 grains of croton chloral hydrate in the fluid drachm, by adding 4 ounces of simple syrup to the above solution, and the disagreeable taste may be removed by any flavoring the pharmacist sees fit to add.—*Druggists' Circular*.

